# Mechanism of Cellular Uptake and Impact of Ferucarbotran on Macrophage Physiology

**DOI:** 10.1371/journal.pone.0025524

**Published:** 2011-09-28

**Authors:** Chung-Yi Yang, Ming-Fong Tai, Chih-Peng Lin, Chen-Wen Lu, Jaw-Lin Wang, Jong-Kai Hsiao, Hon-Man Liu

**Affiliations:** 1 Institute of Biomedical Engineering, National Taiwan University, Taipei, Taiwan; 2 Department of Medical Imaging, National Taiwan University Hospital and College of Medicine, Taipei, Taiwan; 3 Department of Physics, National Tsing Hua University, Hsinchu, Taiwan; 4 Department of Anesthesiology, National Taiwan University Hospital and College of Medicine, Taipei, Taiwan; 5 Department of Medical Imaging, Buddhist Tzu-Chi General Hospital, Taipei Branch, New Taipei City, Taiwan; George Mason University, United States of America

## Abstract

Superparamagnetic iron oxide (SPIO) nanoparticles are contrast agents used for magnetic resonance imaging. Ferucarbotran is a clinically approved SPIO-coated carboxydextran with a diameter of about 45–60 nm. We investigated the mechanism of cellular uptake of Ferucarbotran with a cell model using the murine macrophage cell line Raw 264.7. We observed a dose-dependent uptake of these SPIO particles by spectrophotometer analysis and also a dose-dependent increase in the granularity of the macrophages as determined by flow cytometry. There was a linear correlation between the side scattering mean value and iron content (P<0.001, R^2^ = 0. 8048). For evaluation of the endocytotic pathway of these ingested SPIO particles, different inhibitors of the endocytotic pathways were employed. There was a significant decrease of side scattering counts in the cells and a less significant change in signal intensity based on magnetic resonance in the phenylarsine oxide-treated macrophages. After labeling with SPIO particles, the macrophages showed an increase in the production of reactive oxygen species at 2, 24, and 48 h; a decrease in mitochondrial membrane potential at 24 h; and an increase in cell proliferation at 24 h. We concluded that Ferucarbotran was internalized into macrophages via the clathrin-mediated pathway and can change the cellular behavior of these cells after labeling.

## Introduction

Superparamagnetic iron oxide (SPIO) nanoparticles with dextran or carboxydextran coating are magnetic particles that have been used as a magnetic resonance imaging (MRI) contrast agent [1]. After intravenous injection, these particles are recognized by macrophages that reside mostly in the liver, bone marrow, and spleen [2], [3]. SPIO has been used as a liver-specific contrast agent for detecting primary and metastatic liver tumors [4]. Ferucarbotran is one of the few SPIO particles that is approved for the use in clinical medical imaging [4]. Ferucarbotran is composed of iron oxide and has a core diameter of 4.2 nm and a polymer coating composed of carboxydextran for prevention of aggregation and sedimentation. The final diameter of this composite is on average 62 nm. A brief description of this nanoparticle is listed in Table 1 [5], [6]. The carboxyl side chain of Ferucarbotran makes it easier for it to be conjugated with cell targeting molecules, such as antibodies or peptides. Different compositions of the coating can contribute to different levels of cell uptake efficiency, as shown in human monocytes (macrophage lineage cells) [6].

**Table 1:** Physical and chemical properties of Ferucarbotran.

	Ferucarbotran	Ferumoxytol
Brand Name	Resovist	Code 7228*
Magnetic properties	Superparamagnetic	Superparamagnetic
Core composition	Fe_2_O_3_ and Fe_3_O_4_	Fe_3_O_4_
Core diameter	4.2 nm	6.76 nm
Coating composition	Carboxydextran	Modified dextran
Relaxivity R1 (mM−1 s−1)	7.2 (1.5 T and 37°C)	38
Relaxivity R2 (mM−1 s−1)	82 (1.5 T and 37°C)	83
Surface charge	Ionic	Anionic
Size	45–60 nm	17–31 nm

*Code 7228 is the code name in the development pipeline (AMAG Pharmaceuticals Inc).

Although SPIO particles have been used for stem cell and immune cell labeling, little is known about their mechanism of cellular uptake [7], [8]. Previously, limited results have revealed that the cellular uptake mechanism of Ferumoxides (a dextran coated non-ionic SPIO particle with a diameter of 80–150 nm) into macrophages is via scavenger receptor type A (SR-A) [9]. Compared to the Ferumoxides, Ferucarbotran is smaller, ionic, and has a different coating. A complete understanding of the effect of particle size, surface composition, surface charge, and the mechanism of internalization is important for the future design of novel nanoparticles for highly efficient cell labeling. We previously investigated nanoparticle-labeled macrophages using flow cytometry and observed increased side scattering counts (SSC) when the cells were treated with an increased concentration of SPIO particles [10]. In this article, we evaluated the mechanism involved in macrophage labeling with Ferucarbotran and the change in cellular behavior that occurred after labeling.

## Materials and Methods

### Cell culture

The macrophage cell line Raw 264.7 (Culture Collection and Research Center, Hsin-Chu, Taiwan) has been widely used in the evaluation of macrophage behavior and nanomedicine [11], [12]. These cells were incubated in Dulbecco's modified Eagle Medium (DMEM) (Cellgro, Herndon, VA, USA) supplemented with 10% heat-inactivated fetal bovine serum (FBS), penicillin (50 U/ml), and streptomycin (0.05 mg/ml). The cells were incubated at 37°C in 5% CO_2_. Different SPIO concentrations of Ferucarbotran and Ferumoxides had been employed in the cellular incubation [6]. Among these, induction of apoptosis was found with ≥500 µg Fe/mL of Ferumoxides, and cell viability was not significantly altered by Ferucarbotran concentrations as high as 2000 µg Fe/mL. For iron content determination, Ferucarbotran was added to the culture medium for 24 h at concentrations of 0, 1, 3, 10, 30, and 100 µg Fe/ml, which corresponded to 0, 0.11, 0.33, 1.1, 3.3, and 11 the times the plasma concentration after intravenous administration of Ferucarbotran at the clinically suggested dosage [6].

### Flow cytometry detection of SPIO particle uptake

To study the mechanism of SPIO particle uptake, macrophages were seeded at 1×10^5^ cells per well in six-well plates and allowed to attach for 24 h. These macrophages were incubated with various inhibitors (Sigma-Aldrich, St. Louis, MO, USA), as described below, and with a suspension of Ferucarbotran (10 µg/mL) for 4 h. The inhibitors were phenylarsine oxide (3 µM), cytochalasin D (3 µM), nocodazole (10 µM), filipin (3 ug/ml), and wortmannin (100 nM) [13]. Treated cells were then washed three times with phosphate-buffered saline (PBS; 137 mM NaCl, 2.68 mM KCl, 10 mM Na_2_HPO4, 1.76 mM KH_2_PO_4_, pH 7.4) and then harvested by trypsinization. After centrifugation, the cell pellet was washed once and resuspended in PBS containing 2% FBS solution. The effects of the inhibitors on cellular SPIO particle uptake were examined using a FACSCalibur flow cytometer and CellQuest Pro software (Becton Dickenson, Mississauga, CA, USA).

### Iron content determination

The iron concentration in macrophages was determined as previously described [6], [14]. Briefly, 1×10^5^ macrophages after a 4 h or 24 h exposure to Ferucarbotran at various concentrations (0, 1, 3, 10, 30, and 100 µg Fe/ml) were dissolved in 1% sodium dodecyl sulfate (SDS) buffer. The solution was then examined by using a spectrophotometer (SpectraMax M5/M5e, Molecular Devices, CA, USA) at a wavelength of 248.3 nm. The Fe concentration was determined by comparing the absorption value of each sample with six standards containing Ferucarbotran with Fe concentrations of 0, 1, 3, 10, 30, and 100 µg Fe/ml.

### Mitochondrial membrane potential determination

The mitochondrial membrane potential (MMP) of macrophages was determined as previously described [11], [15]. Briefly, 1×10^5^ macrophages were seeded in a 12-well plate for 12 h and incubated with Ferucarbotran for another 24 h. Cells were harvested and incubated with 3,3′-dihexyloxacarbocyanine (DiOC_6_ [3]), a positively charged dye, at 37°C in a humidified atmosphere of 5% CO_2_ for 30 min. The cells were then washed, centrifuged, and resuspended in PBS. The fluorescent intensities of the cells were measured and analyzed by flow cytometry (BD Biosciences, Franklin Lakes, NJ, USA). Only living cells were included in the analysis.

### Reactive oxygen species determination

Reactive oxygen species (ROS) production after administration of iron oxide was measured using a dichloro fluorescein diacetate (DCFDA) fluorescent probe (Molecular Probes, Eugene, OR, USA) [16]. Briefly, macrophages were exposed to SPIO particles at 100 µg Fe/mL for 0, 15, 60, or 120 min and then washed once with PBS. A total of 1 ml of DMEM and 10 µL DCFDA (10 mM) were added to each well. The cells were then incubated at 37°C for 2 h and then washed with PBS three times and harvested. The fluorescent intensities were measured and quantified by flow cytometry.

### Cell viability assays

Cell viability was evaluated by MTT (3-[4,5-dimethylthiazol-2-yl]-2,5- diphenyltetrazolium bromide) assay. SPIO particle-labeled macrophages and unlabeled cells were grown in triplicate in 24-well plates at 5×10^3^ cells/well for 24 h. Afterward, MTT was added to the medium at a final concentration of 0.5 mg/ml, and the cells were then incubated for 1 h at 37°C in 5% CO_2_. After incubation, the dark-blue formazan dye generated by the living cells was proportional to the number of live cells, and the absorbance at 570 nm was measured using a microplate reader (Infinite F200; Tecan, Männedorf, Switzerland) [17]. MTT data were expressed as the percentage of the average values of the control cells.

### Magnetic resonance imaging

MRI was performed using a clinical 1.5 T MR System (Signa Excite; GE Healthcare Bio-Science, Piscataway, NJ, USA). The cell samples were centrifuged and placed in a water tank, and the tank was then placed in an 8-channel head coil. Two dimension T2-weighted gradient echo pulse (GRE) sequences were used (TR/TE = 550/15 ms, FA = 15). The slice thickness was 1.4 mm with a 0.03 mm gap, and the field of view (FOV) was 14×10.5 cm. Total scan time was 4 min and 43 sec at a number of 3 excitations (NEX). Both axial and coronal planes were scanned for confirmation of the data. The images were then analyzed at a workstation provided by GE Healthcare (Piscataway, NJ, USA).

### Statistical analysis

All experiments were repeated at least twice, and each sample was measured in triplicate. The intracellular iron concentration and SSC mean values were compared using Student's t-test. Statistical significance was assigned if the p value was less than 0.05. All computations were done using SPSS 10.0 software (SPSS Inc, Chicago, IL, USA).

## Results

### SPIO uptake

**Figure 1:**
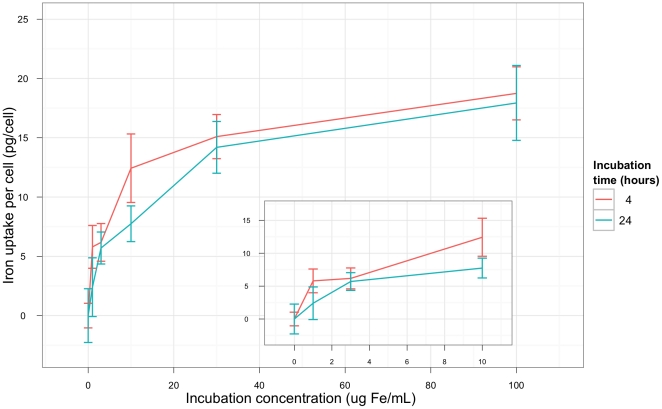
Iron concentrations in macrophages treated with SPIO particles. The amount of SPIO particle uptake by macrophages was measured using a spectrophotometer. Data are presented as mean ± SEM, n = 3. The basal iron content (of the control macrophages) was subtracted from the values. The incubation concentration of SPIO has a strong influence on the amount of particle uptake. There was some (but not significant) increase in intracellular SPIO particle uptake following a long incubation period (24 h) compared to a shorter one (6 h). The maximum particle uptake was observed at 100 µg Fe/ml SPIO, which was statistically significant when compared to the control.

After either a 6- or 24-h incubation with SPIO particles at various concentrations, the macrophages exhibited both increased SSC, as based on flow cytometry, and uptake of iron, as detected by spectrophotometry (Figure 1). The incubation concentration of the SPIO particles had a strong influence on the level of intracellular particle uptake. Incubation for 24 h resulted in a slight but not significant increase in iron uptake compared to the 6 h incubation. In this experiment, the maximum particle uptake was 18 pg Fe/cell. This occurred at a concentration of 100 µg Fe/ml SPIO following a 24 h incubation, which was statistically significant compared to the control.

**Figure 2:**
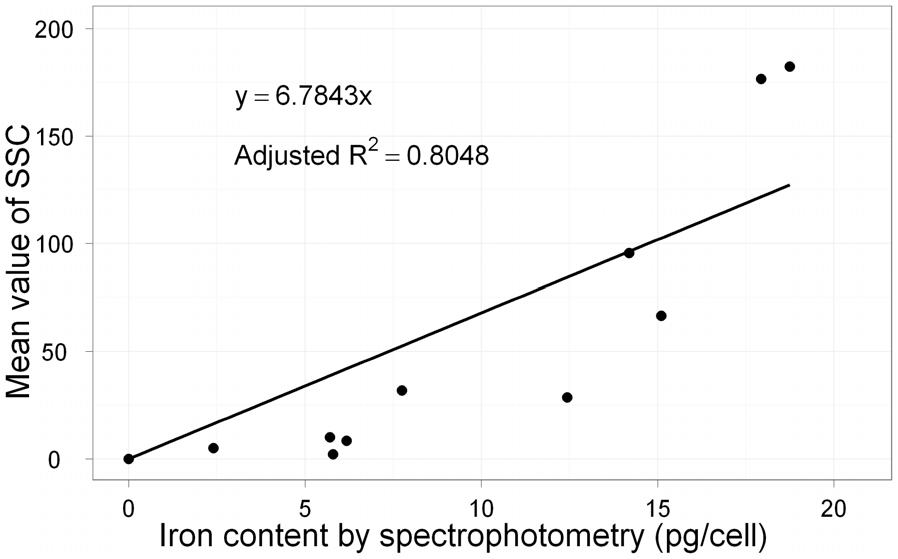
Linear correlation between iron content, as determined using a spectrophotometer, and the mean value of side scattering counts (SSCs), as measured by flow cytometry. Basal iron content and SSC were subtracted. There was a strong correlation between these two factors (P<0.001, R^2^ = 0.8048).

We further investigated the relation between intracellular iron content and side scattering mean values (Figure 2). There was a linear correlation between the side scattering mean value and iron content (P<0.001, R^2^ = 0. 8048). This finding suggested that flow cytometry might be a good indicator for determining intracellular iron content.

### Mechanism of uptake

**Figure 3:**
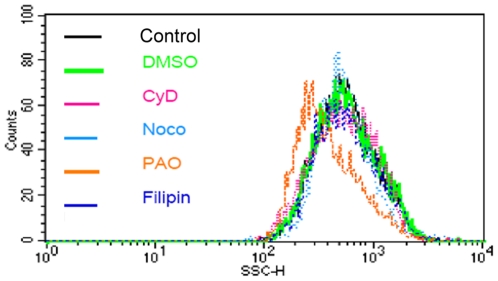
The endocytotic pathway of SPIO particle uptake in macrophages was measured by SSCs via flow cytometry after the cells were treated with different endocytotic inhibitors. There was a left shift in the cell distribution following treatment with the clathrin inhibitor phenylarsine oxide prior to SPIO particle incubation. The other inhibitors tested showed no effect on cellular granularity. This finding suggested that the clathrin receptor plays a major role in the mechanism of cellular uptake of SPIO particles.

**Figure 4:**
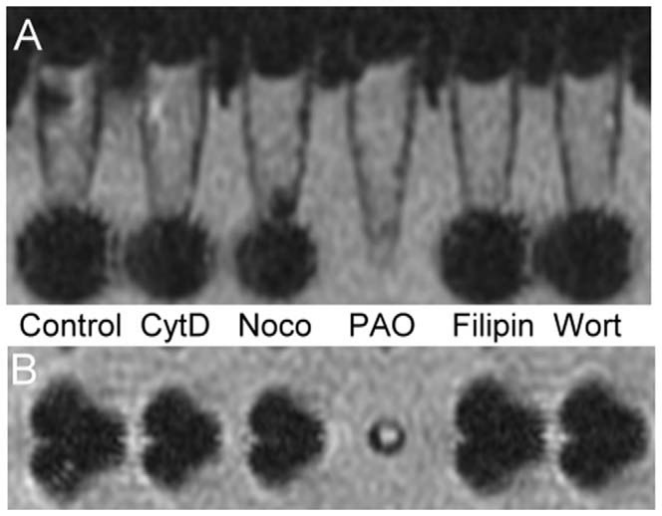
MRI of macrophages treated with different endocytotic inhibitors prior to SPIO particle uptake. (A) Coronal view and (B) axial view under T2 weighted scanning protocol. Cells were centrifuged to the bottom of the test tube and imaged as dark signals. The coronal view and axial view of these cells are consistent. There was a drop in signal intensity in the cells treated with Ferucarbotran and inhibitors, including cytochalasin D (CytD), nocodazole (Noco), filipin, and wortmannin (Wort). In contrast, the signal intensity of the cells treated with phenylarsine oxide (PAO) revealed no loss in signal intensity.

Under flow cytometry, the macrophages pretreated with phenylarsine oxide exhibited decreased SSC compared to the control. In contrast, no significant change in SSC was found in the cytochalasine D, nocodazole, filipin, or wortmannin treated macrophages (Figure 3). The endocytotic pathway of macrophages involved in SPIO particle uptake was also confirmed by our MRI results (Figure 4). Control Ferucarbotran-labeled cells showed a decreased signal intensity under T2 weighted images, and those labeled cells pretreated with cytochalasine D, nocodazole, filipin, or wortmannin demonstrated a similar drop in signal. However, macrophages incubated with phenylarsine oxide had lower change in signal intensity compared to these other groups.

### Cellular behavior

**Figure 5:**
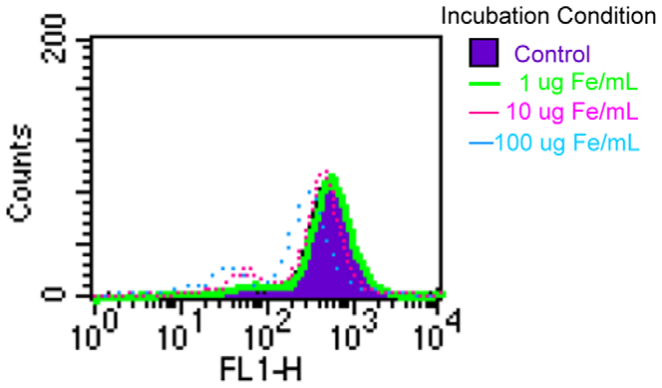
The MMP of macrophages treated with SPIO particles was measured by fluorescent intensity of the FL-1 channel. There was a leftward shift of the fluorescent intensity after the cells had ingested the SPIO particles, and this response occurred in a dose-dependent manner.

**Figure 6:**
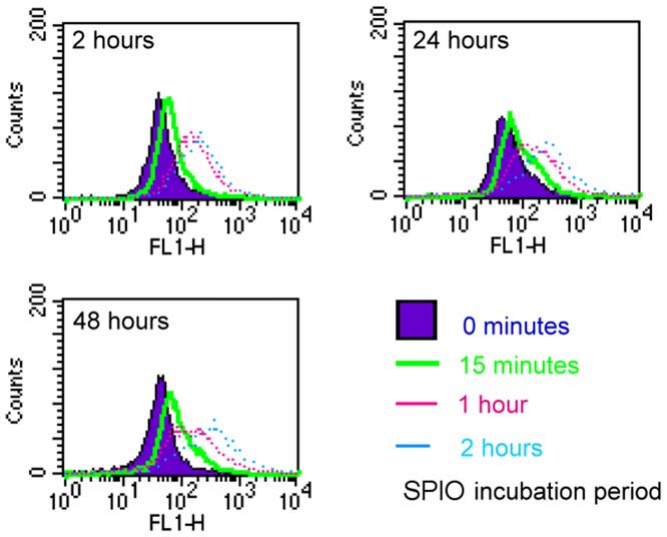
Flow cytometry measurement of ROS production in macrophages after SPIO particle uptake at different time points. The intensity of the fluorescence of the FL-1 channel was linearly correlated to ROS production. ROS increased when the cells were exposed to SPIO particles after 2 h, and this effect lasted up to 48 h. There was also a dose-dependent response when the cells were incubated with SPIO particles for longer periods, indicating that ROS production is related to particle loading.

**Figure 7:**
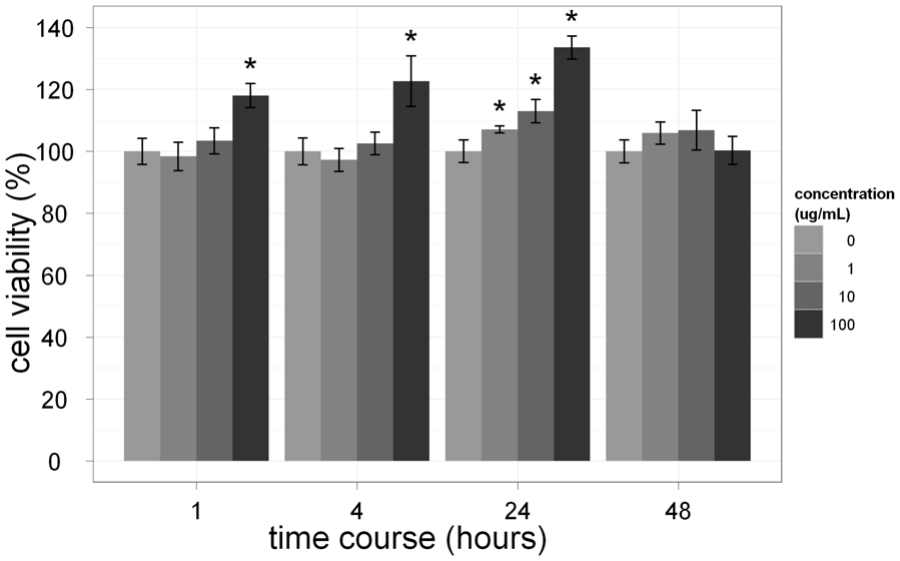
Macrophages were incubated in the absence (control) or presence of 1, 10, and 100 µg/ml of SPIO particles for 1, 4, 24, and 48 h. The effect of SPIO particles on cell viability was determined via MTT assays immediately after incubation. Data are expressed as the mean and error of three determinations, with each assay performed in triplicate. Dunnett's test treats one group as a control and compares all other groups against it. *p<0.05.

The evaluation of cellular behavior was performed by MMP assay, ROS assay, and MTT assay (Figure 5, 6, 7). There was a dose-dependent decrease in MMP in the cells treated with Ferucarbotran for 24 h at the concentrations of 1, 10, and 100 µg Fe/mL. The ROS production increased after 2 h and lasted up to 48 h. We also observed a dose-dependent effect on ROS production. Macrophages treated with 1 µg Fe/mL of Ferucarbotran produce less ROS compared to the cells exposed to 100 µg Fe/mL. In regards to the MTT assays, macrophages labeled with 100 µg Fe/mL Ferucarbotran exhibited a significant increase in proliferation at 1, 4, and 24 h; however, this change was not observed at 48 h. Macrophages treated with 1 and 10 µg Fe/mL did not show any change in cell proliferation until the 24 h time point.

## Discussion

### SPIO particle uptake

The findings in this study illustrated the dose-dependent manner of SPIO particle uptake at lower SPIO concentrations (1 to 100 µg Fe/ml), which is characteristic of endocytotic phenomenon in cells. These findings are also compatible with the results observed when peritoneal macrophages are treated with dextran-coated SPIO particles [9]. However, a time-dependent manner of intracellular SPIO particle uptake was not seen in this study. Dose- and time-dependent SPIO particle uptake in macrophages has been reported from 30 min to 6 h (at 100 µg Fe/mL) and at higher concentrations ranging from 30 to 1000 µg Fe/mL during a 1 h incubation [18]. In this previous study, the iron uptake of macrophages approached a constant level with a >200 min exposure at a fixed concentration (100 µg Fe/mL) or with >700 µg Fe/mL after a 1 h incubation period. Therefore, the saturation of iron uptake after a >200 min incubation period could explain why there was only a minimal increase in iron uptake after a 24-hr incubation in this current study. This time-dependent saturation suggested that a prolonged incubation is not necessary for improving the amount of iron uptake. Besides, under physiologic conditions, intravenously administered Ferucarbotran has a short half-life of about 3.9–5.8 min for the initial fast phase (accounting for about 80% of the initial concentration) and about 2.4–3.6 h for the second phase. Furthermore, a prolonged exposure of macrophages to SPIO has been shown to be cytotoxic [18], [19]. Therefore, to avoid unnecessary side effects, an appropriate selection of incubation period should be made.

Our data revealed a significantly higher cell iron content when the cells were treated with carboxydextran-coated SPIO particles compared to previous results using dextran-coated SPIO particles. Although different measuring methods (spectrophotometer versus inductively coupled plasma atomic emission spectroscopy) and cell types (macrophage cell line versus peritoneal macrophages) were used in these studies, our inference is supported by previous results [6] that revealed a 2–3 fold increase in SPIO particle uptake in the carboxydextran group relative to the dextran group.

The increase in iron uptake correlated with an increase in SSC as measured by flow cytometry. This finding was consistent with our previous work [10]. This increase in SSC likely results from increased granularity when the cells ingest these nanoparticles.

There are advantages of measuring cells with flow cytometry when compared to spectrophotometer or inductively coupled plasma mass spectrometry. First, flow cytometry measures the cell content without physical damage to the cells, which can occur when using either a spectrophotometer or ICP-MASS. As such, cell morphological data can subsequently be acquired when flow cytometry is used. Second, with flow cytometry, many other kinds of cell data (e.g., individual cell diameter) can also be obtained. Third, individual cell data can be combined for statistical analysis when the targeted cells are heterogeneous. Thereafter, we propose this method might be used for semi-quantitation of internalization of nanoparticles by cells.

### Mechanism of uptake

Evidence indicates that lysosomal metabolism takes place after internalization of SPIO particles into cells [1], [20], [21], [22]. Endocytosis of foreign materials can be divided into phagocytosis for larger foreign materials and pinocytosis for soluble material or nanoparticles. Pinocytosis can be subdivided into clathrin-mediated endocytosis (100–150 nm), caveolae (50–80 nm), and macropinocytosis (500–2000 nm). Phagocytosis occurs in specialized cells when the foreign materials are larger than 750 nm [23], [24]. The major route for endocytosis is clathrin-dependent endocytosis, which is found in virtually all cells. Ferucarbotran is a nanoparticle, and its hydrodynamic size could be moderately larger with increasing SPIO concentration (81±2.05 nm at 100 µg Fe/ml) [25]. Therefore it is possible that Ferucarbotran is internalized by cells via the pinocytosis pathway.

To determine which endocytotic pathway is involved when macrophages encounter carboxydextran-coated SPIO particles, several inhibitors related to different endocytotic pathways were incubated separately with macrophages prior to SPIO treatment. These inhibitors included cytochalasine D, which blocks actin dependent process such as macropinocytosis; nocodazole, which inhibits microtubule function involved in intracellular vesicle trafficking; phenylarsine oxide, which inhibits the clathrin-mediated endocytotic pathway; filipin, which disturbs caveolae pathways; and wortmannin, which interferes with phosphoinositol 3-kinase involved in macropinocytosis.

Despite that the hydrodynamic size of Ferucarbotran is relatively small (81±2.05 nm at 100 µg Fe/ml), our results revealed the internalization of carboxydextran coated and ionic particles by macrophages occurred via the clathrin-mediated pathway. It is believed that the clathrin receptor contributes to internalization of many kinds of nanoparticles [13]; however, the correlation between SPIO factors (such as size, coating, and surface charge) and the endocytotic pathways is not well understood. Further evaluation of different SPIO particles of various sizes or coatings and their interaction with a particular cell type would clarify these important relationships.

The MRI findings in this study were consistent with the flow cytometry data. The SSC measurements obtained by flow cytometry not only provided information related to the intracellular uptake of SPIO particles, but it also imply of the efficacy of the MRI signal production of the labeled cells.

### Cellular behavior

Our observations suggested that the initial degradation of these particles began within days, since there was an apparent elevation in both ROS and MMP as early as 24 h. It has been reported that no increase in ROS occurs in mesenchymal stem cells, HeLa cells, or macrophages when they encounter dextran-coated SPIO particles. However, fluctuations in baseline ROS production during different time points makes the data difficult to interpret [16]. The discrepancy between our results and others might be due to the different cell lines used. The macrophages we used exhibit strong phagocytic, migratory, and cytokine secretion behaviors, which indicated their active role in the eradication of foreign materials and invading organisms [11]. Consequently, the degradation of SPIO particles in these macrophages is likely very active and fast compared to other cells. Similar to that observed in mesenchymal stem cells, the increased proliferation of macrophage at 24 h of incubation may have been due to Ferucarbotran diminishing intracellular H_2_O_2_ via its intrinsic peroxidase-like activity and also accelerated cell-cycle progression, which may have been mediated by the free Fe released following lysosomal degradation and subsequent alteration in the expression of protein regulators of the cell cycle [17].

The maintenance of the MMP is crucial for energy production in the form of ATP synthesis. The loss of MMP increases the release of cytochrome c and triggers a series of intracellular events, which ultimately result in apoptosis. Thus, loss of MMP is usually considered an early indicator of apoptosis [26]. Our study revealed that at 10 µg Fe/mL (equivalent to the plasma concentration following intravenous administration of Ferucarbotran) there was a mild decrease in the MMP and a larger decrease at 100 µg Fe/mL (Figure 5). The MTT assays revealed no significant toxicity in the Ferucarbotran-labeled macrophages at the various concentrations up to 48 h (Figure 7). In a previous study the induction of apoptosis was only observed in monocytes treated with ≥500 µg Fe/mL of Ferumoxides, but this did not occur in those cells treated with Ferucarbotran up to 2000 µg Fe/mL [6]. However, delayed cell death was shown in macrophages treated with Ferucarbotran [19]. Although there is the concern of cytotoxicity with SPIO and/or ultrasmall superparamagnetic iron oxide (USPIO) particles toward macrophages, especially after prolonged exposure, such processes could likely be antagonized by use of a therapeutic radical scavenger [18], [19].

Because of their smaller size, USPIO particles accumulate in the reticuloendothelial system much slower than SPIO particles, which results in a longer plasma half-life. While SPIO particles mostly accumulate in the reticuloendothelial system, USPIO particles can enter into the interstitial space from capillaries by transcytosis and then into the lymph nodes via the lymphatic vessels [27]. Different cellular uptake efficiencies between SPIO and USPIO particles have also been previously observed [6]. It has also been shown that prolonged exposure to USPIO particles results in decreased cell viability in macrophages [18], [19]. Given its smaller particle size, different charge, and distinct surface properties (coating), it is possible that the cellular uptake of USPIO particles occurs via a different endocytosis pathway (e.g., caveolae); however, this requires further verification in future studies.

### *In vivo* cell tracking

Macrophage/monocyte labeling for in vivo cell tracking could be potentially applied to specific targeting of inflammatory processes, such as tumor localization, atherosclerotic plaques, or infections. Several possible noninvasive approaches including optical imaging, nuclear imaging, and MRI have been reported [28], [29], [30], [31], [32], [33], [34], [35], [36], [37]. Though nuclear imaging has good sensitivity, the use of radionuclides introduces potential radiation damage to the cells and the overall spatial resolution is relatively poor. Optical imaging is highly sensitive, inexpensive, and radiation free. This method has been successfully used for targeting the peritumoral inflammatory response in a murine model [37]; however, many cyanine fluorescent dyes are cytotoxic, and their fluorescence can not penetrate soft tissues enough to be detected by optical sensors. That is, if the imaging target is deep in the human body, it is unlikely to be detected with a small dose of fluorescent dye. Compared to other techniques, the sensitivity of MRI is not as good as nuclear or optical imaging; however, MRI has outstanding spatial resolution, soft tissue contrast, and signal-to-noise ratio (SNR). Many approaches, including development of novel paramagnetic materials, intracellular labeling, or amplification of signals produced by contrast mediums, have been investigated. For example, polymerization of paramagnetic substrates has been proposed as an MR signal amplification technique [38], and strong MR signal enhancement by a threefold increase in relaxivity makes it possible to identify cell surface receptor expression in vitro. It is also possible to use this technique in conjunction with SPIO or USPIO particles to augment the MR signal for identification of different molecular targets in vivo; however, this method could change the endocytosis pathway because of the significant increase of the size of the polymer. Further investigation of the mechanism of cellular uptake and the evaluation of biocompatibility are necessary in future studies.

### Limitations

Currently, Ferucarbotran was not on market since 2009 due to marketing strategy; thus, access to this particular iron oxide compound is limited. However, its superior labeling efficiency and MRI signal production make Ferucarbotran an attractive option for cellular imaging.

### Conclusions

The endocytotic mechanism of SPIO uptake into macrophage was determined to be via the clathrin-mediated pathway. Further development of nanoparticles targeting the clathrin receptor may increase the efficiency of this cell labeling method. Moreover, we observed significant cellular changes in macrophages after SPIO particle labeling. Finally, we developed a strategy for evaluating cellular uptake of SPIO particles by measuring SSC using flow cytometry, which demonstrated a high correlation with the results of both spectrophotometric and MRI methods.
